# The Secret Life of Viral Entry Glycoproteins: Moonlighting in Immune Evasion

**DOI:** 10.1371/journal.ppat.1003258

**Published:** 2013-05-16

**Authors:** Jonathan D. Cook, Jeffrey E. Lee

**Affiliations:** Department of Laboratory Medicine and Pathobiology, Faculty of Medicine, University of Toronto, Toronto, Ontario, Canada; Columbia University, United States of America

Survival of infection with Ebola virus (EBOV) depends on the ability of the host to mount early and strong immune responses [1], [2]. However, given that EBOV cases are associated with 40%–90% human mortality, EBOV has developed intricate solutions to human immunological defenses. Enveloped viruses, like EBOV, must deposit their genetic material within a cell to ensure their propagation. The roles of viral envelope glycoproteins in mediating virus attachment to host cells and catalyzing the subsequent fusion of the viral and host plasma membranes have been well described (reviewed in [3]). Given the limited number of genes in EBOV and other viruses, it stands to reason that these conformationally labile glycoproteins are also involved in more than just the initial steps of a productive infection. There is strong evidence that viral entry glycoproteins (GP) are modulators of host antiviral defenses (Table 1). In this article, we discuss our current structural understanding of the functions of envelope entry glycoproteins in immune evasion using EBOV as our example.

**Table 1:** Viral entry glycoprotein-mediated immune evasion strategies in other viral families.

Viral Family	Immune Evasion Mechanism	Examples/Comments	Ref
*Arenaviridae*	Glycoprotein shedding/secretion	Lassa virus: shed GP1-mediated immune evasion has been attributed to differential glycosylation of the shed and transmembrane glycoprotein complex.	[37], [38]
*Coronaviridae*	Direct humoral antagonism	SARS CoV: spike protein acts as a ligand for phenotypic conversion of B cells into macrophage-like cells.	[39]
*Filoviridae*	see article		
*Flaviviridae*	Glycan shielding	The hepatitis C virus E1/E2 glycoprotein escapes neutralizing antibodies in a glycoprotein-dependent manner.	[40], [41]
	NK and innate immune antagonism	The hepatitis C virus E2 glycoprotein binds CD81 and blocks natural killer cell activation.	[42]
*Herpesviridae*	Glycan shielding	Bovine herpes virus gp180 O-linked glycans shield against humoral assault and are conserved across all gammaherpesvirus gp350 homologs.	[43]
	Antigen presentation antagonism	Epstein Barr virus sgp42 binds MHC class II, thereby interfering with antigen presentation to CD4^+^ T-cells.	[44]
*Orthomyxoviridae*	Glycan shielding	Glycans present on the Influenza A virus HA glycoprotein protect temporally diverse pandemic strains in a conserved manner.	[45], [46]
*Paramyxoviridae*	Glycoprotein shedding/secretion	Respiratory syncytial virus G glycoprotein acts both as a decoy for host antibodies and can modulate immunity via immune receptor interactions.	[47]
	Glycan shielding	Nipah virus F protein contains N-linked glycans that offer a protective role against the host antibody response.	[48]
*Retroviridae*	Glycoprotein shedding/secretion	HIV-1 gp120 shedding competes with the gp160 complex for host antibodies.	[49]
	Immunosuppressive structural motif	Peptides derived from HIV-1 gp41 inhibit T-cell activation.	[19]
	Immunosuppressive structural motif	HTLV-1 gp21 immunomodulatory region inhibits IgG response by ∼40 fold when compared to mutant recombinant protein.	[50]
	Glycan shielding	HIV-1 gp120 glycan shield protects otherwise neutralizing epitopes from humoral antagonism and directs antibodies towards variable loops.	[7], [51]
	Direct innate immunity antagonism	HIV-2 *env*-encoded glycoprotein counteracts BST-2-mediated viral tethering.	[32]
	Antigen presentation antagonism	HIV-1 gp41 can interrupt TCR-CD3 interactions to modulate T-cell proliferation.	[52]
	Antigen presentation antagonism	HIV-1 gp41 interacts with HLA-associated invariant chain and may have a role in MHC-directed antagonism.	[28], [53]

## How Does Glycosylation of Ebola Virus Envelope Proteins Facilitate Immune Evasion?

**Figure 1:**
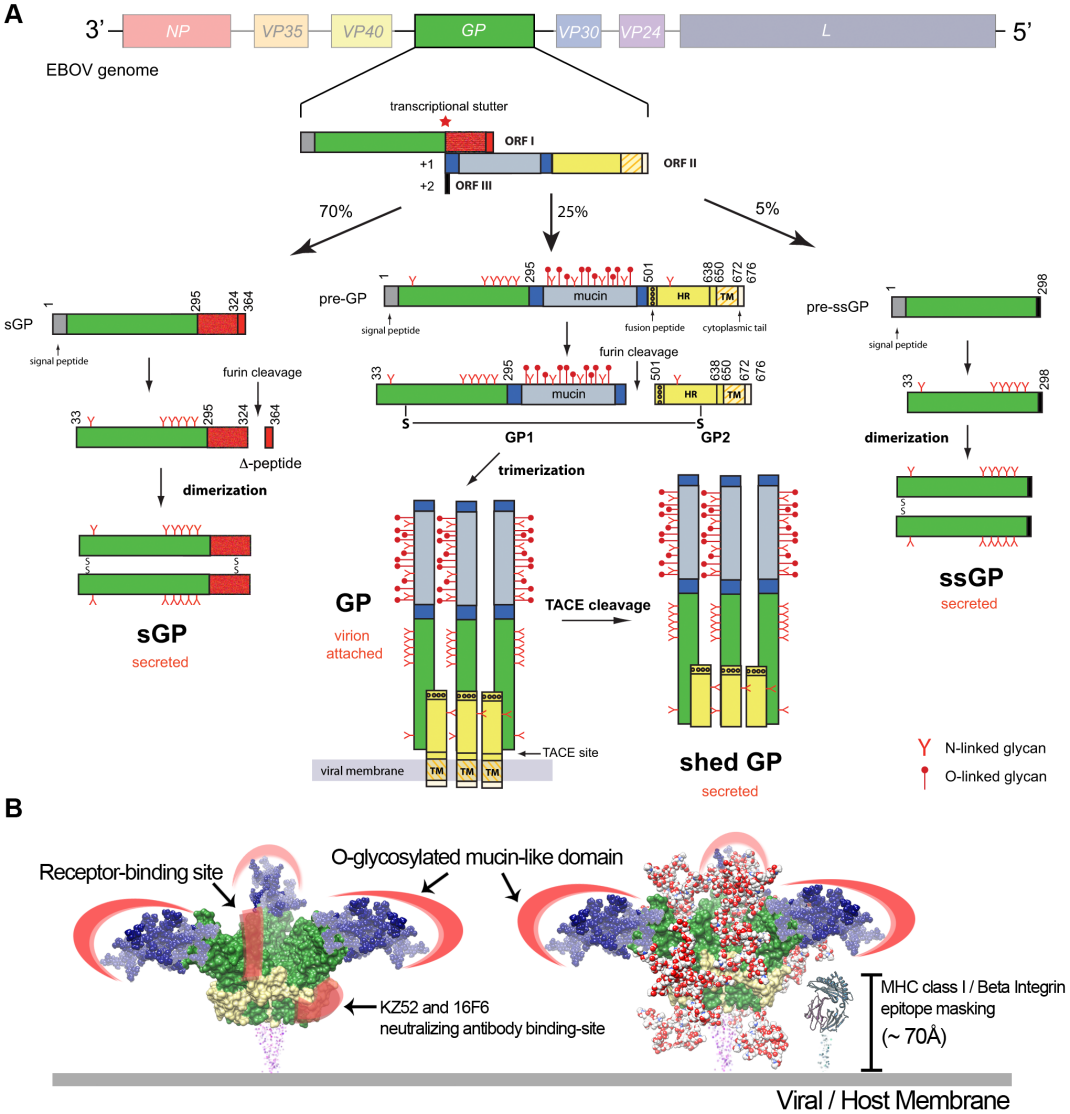
Ebola virus glycoproteins. (A) Processing of EBOV glycoproteins. The EBOV genome contains seven genes (3′-*NP-VP35-VP40-GP-VP30-VP24-L*-5′), but nine proteins are produced due to editing events in the *GP* gene. The *GP* gene primary transcript encodes for a ∼110 kDa, dimeric secreted GP (pre-sGP). Furin cleavage of pre-sGP produces mature sGP and a secreted Δ-peptide. Transcriptional stuttering results in the production of the envelope-attached GP and a small, secreted GP (ssGP). The GP is the only virally encoded protein on the EBOV surface and is cleaved by furin to form a disulfide-linked GP1-GP2 heterodimer, which then assembles as trimers on the virus surface. GP1 contains the receptor-binding site for host cell attachment, while GP2 contains a helical heptad-repeat (HR) region, transmembrane anchor (TM), and a 4-residue cytoplasmic tail. A cleavage at the membrane-proximal external region by the tumor necrosis factor-α converting enzyme (TACE) releases the shed GP. The first 295 residues of ssGP, sGP, and GP are common, but each protein has a different C-terminus, leading to different functions. (B) Epitope masking by EBOV glycoproteins. Molecular surface of EBOV GP subunits (PDB code: 3CSY) are shown in green (GP1) and yellow (GP2). Complex-type N-linked glycans are modeled onto the EBOV GP surface as red/white spheres to reveal a heavy glycan layer that buries much of the GP surface, including the receptor-binding site; only a small patch at the base of the GP is accessible (KZ52/16F6 antibody-binding site). The O-linked glycosylated mucin-like domain (blue) is modeled onto EBOV GP, and thought to form an extended structure that provides another glycan layer of protection to the virus. EBOV GP is estimated to be ∼150 Å in height. Given the size and shape of EBOV GP, smaller cellular surface proteins, such as MHC class I and β-integrins (∼70 Å in height), may be sterically blocked.

In EBOV, four variants of the envelope glycoprotein are synthesized as a result of transcriptional stuttering or post-translational processing (Figure 1A). About 25% of transcripts from the *GP* gene produce the virion-attached or envelope spike GP that is important for entry. The surface of the envelope GP is covered with N- and O-linked glycans. Depending on the EBOV species, the envelope GP contains 11–18 N-linked glycan sites. Furthermore, EBOV GP contains an unstructured ∼150-residue mucin-like domain that is heavily modified with O-linked glycans (∼80 sites) [4]. The N-linked glycans are a heterogeneous mixture of ∼60 different species of high-mannose, hybrid, and complex oligosaccharides, while the O-linked glycans consist of primarily smaller trisaccharide structures (core 2) that contain varying amounts of sialic acids [5].

Epitope masking is a major mechanism of viral immune evasion. Modeling of the EBOV GP core structure reveals a surface covered in oligosaccharides (Figure 1B). The dense clustering of glycans creates an unfavorable environment for the interaction of otherwise neutralizing antibodies. Moreover, critical regions on EBOV GP, such as the receptor-binding site, are hidden under layers of glycan. No antibodies have been identified to target the receptor-binding site, however a number of neutralizing antibodies have been generated against the more variable mucin-like domain [6]. The mucin-like domain is not necessary for EBOV entry [4]. Essentially, the EBOV GP glycans direct the immune system to produce antibodies against highly variable or dispensable regions on the viral surface. This also occurs in hosts infected with HIV-1; nonbroadly neutralizing antibodies are generated against the variable V1/V2/V3 loops [7]. In mice, removal of the mucin-like domain of the EBOV GP leads to the production of cross-species antibodies directed at the conserved glycoprotein core structure [8]. A small area near the base of the EBOV GP core is available to immune surveillance (Figure 1B). This nonglycosylated patch on GP is conserved in both Zaire and Sudan EBOV species, and the neutralizing antibodies KZ52 and 16F6-1 bind to this hotspot [9]. However, given its close proximity to the viral membrane and the density of GP spikes on the surface, it is not clear how accessible this epitope is.

EBOV GP also has the unique ability to mask the function of host cellular proteins important in response to viral pathogens. Transient expression of EBOV GP results in low detectable levels of various cell surface proteins such as major histocompatibility complex (MHC) class I proteins and several members of the β-integrin family [10], [11]. Initially, it was thought that EBOV GP downregulated expression or degraded these proteins from the cell surface. However, MHC class I and β-integrins are not removed from the cell surface. Rather, the mucin-like domain of EBOV GP provides a “glycan umbrella” that shields surface epitopes and inhibits surface protein recognition [11]–[13] (Figure 1B). This represents a novel mechanism of disrupting immune function that does not involve downregulation or degradation of surface proteins.

## What Roles Do Shed Viral Glycoproteins Play in Immune Evasion?

The shedding or secretion of soluble viral glycoproteins exemplifies another viral strategy of humoral misdirection. Many enveloped viruses, including EBOV, Lassa, respiratory syncytial, herpes simplex, and HIV-1, generate free glycoproteins that act as either “antibody sinks” or decoys of host immunity (Table 1). EBOV-infected cells secrete two glycoproteins (secreted GP and shed GP) into an infected person's sera [14], [15]. Most of the transcripts (70%) for the *GP* gene encode the 110-kDa, dimeric, secreted GP (sGP) (Figure 1A). A cleavage at the membrane-proximal external region by the tumor necrosis factor-α converting enzyme (TACE) releases the trimeric glycoprotein, termed shed GP. In 5% of the transcripts, insertion of two adenosines produces a small 298-residue secreted GP (ssGP), of unknown function. The first 295 amino acids of sGP are common with the envelope GP, but due to transcriptional stuttering, the sGP C-terminus forms different disulfide linkages leading to a homodimeric rather than a trimeric assembly (Figure 1A). As a result, sGP lacks regions found in GP that have been shown to be important in the neutralization of the virus [9]. sGP and shed GP likely compete with virion-attached GP for antibody binding [16]. Most of the antibodies derived from EBOV survivors or macaques are directed towards sGP rather than the virion-attached GP [17], [18]. Antibodies that bind to sGP or shed GP are likely nonneutralizing, and those neutralizing antibodies that cross-react between sGP and GP may be absorbed by the much more abundant sGP. In a guinea pig model of EBOV infection, shed GP inhibits the neutralizing activity of EBOV antibodies [15].

## How Do Viral Glycoproteins Actively Suppress Host Immunity?

**Figure 2:**
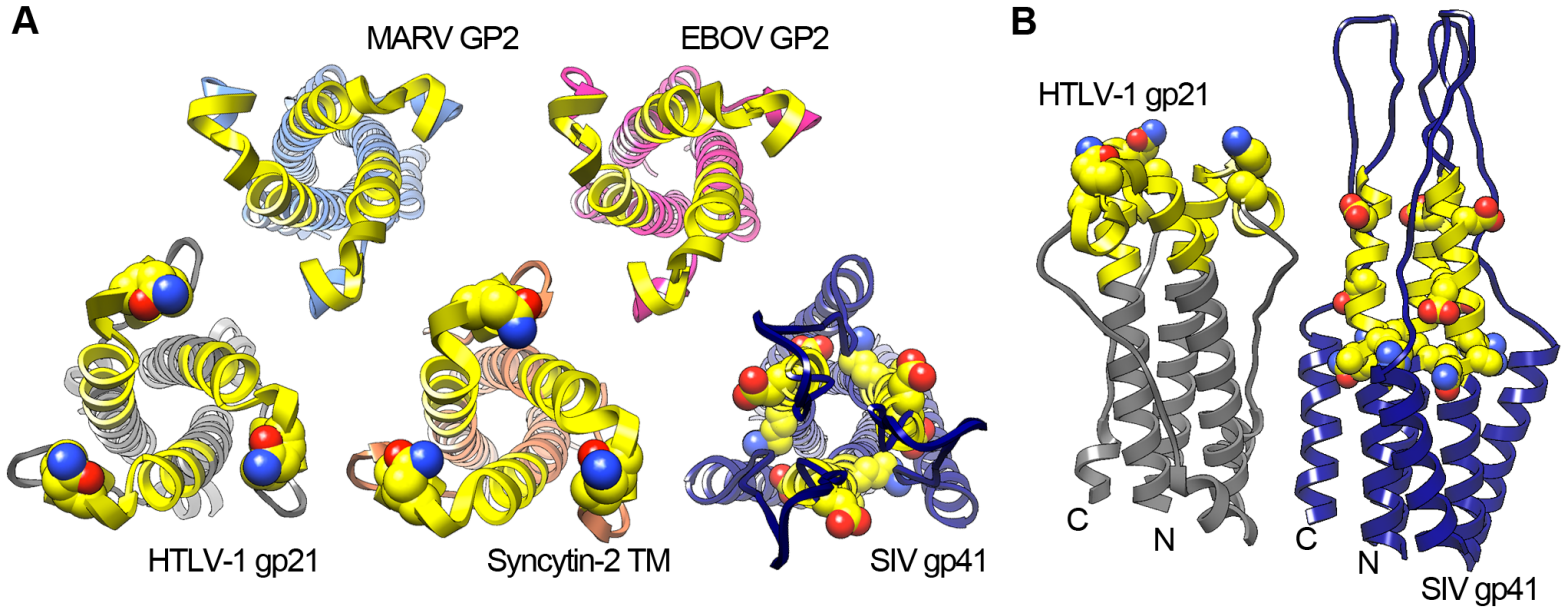
Structural conservation of the viral glycoprotein immunomodulatory region. The immunomodulatory region is approximately 20 amino acids long and is found within numerous retroviruses and filoviruses. In each case presented here, the experimentally defined immunomodulatory region is rendered in yellow and residues that are necessary for the observed immunomodulatory activity are depicted as spheres. (A) Head-on view of viral glycoproteins exhibiting a conserved three-fold pinwheel structure. (B) Side-view illustrating the differences in possible interaction faces between the lentiviral gp41 immunomodulatory region and a representative retrovirus, HTLV-1. The outward positioning of the important immunomodulatory residues shown for HTLV-1 gp21 can be observed in all available retroviral and filoviral post-fusion glycoprotein structures except SIV gp41. The PDB codes for the fusion glycoproteins are as follows: EBOV GP2, 2EBO; MARV GP2, 4G2K; HTLV-1 gp21, 1MG1; syncytin-2, 1Y4M; SIV gp41, 2EZO.

In a seminal paper, Cianciolo et al. described immunomodulation by a synthetic peptide derived from the fusion subunit of the HIV-1 envelope glycoprotein [19]. The immunomodulatory region (IR) is comprised of a disulfide-linked loop situated between the heptad-repeat regions of the fusion subunit, and similar structures have been identified in numerous retroviruses and filoviruses (Figure 2A). Point mutations introduced into the IR of HIV-1 gp41 abrogate the modulation of host cytokine expression in vitro and increase antibody responses in rats immunized with mutant protein [20]. Synthetic peptides derived from the IR regions of GP2 of Ebola and Marburg viruses inhibit the expression of IFN-γ, IL-2, and IL-10, lower CD4^+^ and CD8^+^ cell activation, and increase immune cell apoptosis [21]. The fusion domain from Moloney murine leukemia virus expressed on various tumor cell lines facilitates xenograph immune evasion and natural killer cell antagonism [22]. Interestingly, the human endogenous retrovirus-derived syncytin-2 retains the immunomodulatory activity associated with the viral envelope glycoproteins, but the closely related syncytin-1 differs in the IR region, ablating this function [23]. These retrovirus-derived syncytin proteins are implicated in both cell–cell fusion during placental development and in maternal–fetal tolerance, clearly pointing to a role in immune evasion [24]. Available structures of the post-fusion glycoprotein subunit show that the disulfide-bonded immunomodulatory motif exists as a conformationally conserved region at the apex of the fusion subunit, with residues identified by mutagenesis as important for immunosuppression displayed outwards (Figure 2B). Interestingly, in the SIV fusion subunit the same region is not found at the apex but rather on the central helical heptad-repeat region. One possible target of the HIV-1 gp41 IR is CD74, a type II single-pass transmembrane protein that, among other functions, chaperones MHC class II dimers from the endoplasmic reticulum to the MHC class II compartment (MIIC) for antigen loading [25], [26], and is also implicated in MHC class I cross-presentation [27]. Recently, it was determined that the ectodomain of human CD74 binds to residues corresponding to a region adjacent to the conserved HIV-1 gp41 IR. When peripheral blood mononuclear cells (PBMCs) were incubated with recombinant post-fusion HIV-1 gp41, an increase in phosphorylated ERK occurred. Furthermore, this activation was inhibited in a dose-dependent manner by treatment with soluble recombinant CD74 ectodomain [28]. The activation of the ERK/MAPK pathway via high levels of the CC-chemokine RANTES (or other exogenous signals) is responsible for increased infectivity of HIV-1 [29]. In support of these findings, siRNA knockdown of CD74 effectively curbs HIV-1 infectivity [30]. Although these works are stimulating, more extensive research is required to generate a complete description of viral fusion glycoprotein-associated immunosuppression. Like HIV-1, the host targets of the immunomodulatory motif found in other species of virus remain poorly defined and await further studies.

## What Are the Innate Restriction Strategies Targeted toward Viral Glycoproteins?

Host strategies for viral restriction are not limited to the humoral arm of the immune system. The interferon-α-induced innate viral restriction factor BST-2 (also called tetherin and CD317) is a common target of viral glycoprotein modulation [31]. As viruses bud from the cell surface, they are coated with a membrane derived from the host cell. As a result, host BST-2 is incorporated in the membrane of the nascent virion and forms a protein tether to prevent viral release. This nonspecific restriction factor potentially plays a protective role against infections due to retroviruses, filoviruses, arenaviruses, flaviviruses, rhabdoviruses, and orthomyxoviruses (Table 1).

EBOV and HIV-2 both downregulate BST-2 by interactions mediated through their respective viral glycoproteins [32], whereas HIV-1 makes use of the accessory protein Vpu to achieve this same outcome. Some viruses degrade BST-2 or sequester it in intracellular compartments. For example, the HIV-2 envelope glycoprotein appears to sequester the constitutively endocytosed BST-2 in transferrin-positive endosomes [33]. Recent studies have shown that EBOV GP does not remove BST-2 from the cellular surface [34] or sequester it in intracellular sites or lipid rafts [35]. EBOV GP interferes with BST-2-mediated virion capture independently of the mucin-like domain, and neither an engineered form of GP lacking the transmembrane domain nor the dimeric sGP antagonize BST-2 restriction [32]. HIV-1 Vpu interacts with BST-2 via a helical interface found within the transmembrane domains of the two proteins [36]. Accordingly, it may be worthwhile to explore the role of the transmembrane domain of EBOV GP in BST-2 antagonism.

## Perspectives

Viruses have developed remarkable mechanisms to inhibit the adaptive and innate immune systems of their hosts. Clearly, viral entry glycoproteins play critical roles in these activities. However, many of these roles and biological pathways are poorly defined. With new infectious diseases emerging and classical viral diseases reemerging, closer examination of viral entry glycoproteins as targets for preventative or therapeutic strategies is warranted.
